# Edge-mediated skyrmion chain and its collective dynamics in a confined geometry

**DOI:** 10.1038/ncomms9504

**Published:** 2015-10-08

**Authors:** Haifeng Du, Renchao Che, Lingyao Kong, Xuebing Zhao, Chiming Jin, Chao Wang, Jiyong Yang, Wei Ning, Runwei Li, Changqing Jin, Xianhui Chen, Jiadong Zang, Yuheng Zhang, Mingliang Tian

**Affiliations:** 1 High Magnetic Field Laboratory, Chinese Academy of Science (CAS), Hefei 230031, China; 2 Laboratory of Advanced Materials, Department of Materials Science, Collaborative Innovation Center of Chemistry for Energy Materials, Fudan University, Shanghai 200438, China; 3 Computational Physics and computational Mechanics, Institute of Fluid Physics, China Academy of Engineering Physics, Mianyang 621900, China; 4 Key Laboratory of Magnetic Materials and Devices, Ningbo Institute of Material Technology and Engineering, Chinese Academy of Sciences, Ningbo 315201, China; 5 Key Laboratory of Extreme Conditions Physics, Beijing National Laboratory for Condensed Matter Physics and Institute of Physics, Chinese Academy of Sciences, Beijing 100190, China; 6 Collaborative Innovation Center of Advanced Microstructures, Nanjing University, Nanjing 210093, China; 7 Hefei National Laboratory for Physical Science at Microscale, Department of Physics, University of Science and Technology of China, Hefei 230022, China; 8 Department of Physics, Materials Science Program, University of New Hampshire, Durham, New Hampshire 03824, USA

## Abstract

The emergence of a topologically nontrivial vortex-like magnetic structure, the magnetic skyrmion, has launched new concepts for memory devices. Extensive studies have theoretically demonstrated the ability to encode information bits by using a chain of skyrmions in one-dimensional nanostripes. Here, we report experimental observation of the skyrmion chain in FeGe nanostripes by using high-resolution Lorentz transmission electron microscopy. Under an applied magnetic field, we observe that the helical ground states with distorted edge spins evolve into individual skyrmions, which assemble in the form of a chain at low field and move collectively into the interior of the nanostripes at elevated fields. Such a skyrmion chain survives even when the width of the nanostripe is much larger than the size of single skyrmion. This discovery demonstrates a way of skyrmion formation through the edge effect, and might, in the long term, shed light on potential applications.

## Abstract (web summary)

The recent experimental creation of magnetic skyrmions, topologically protected particle-like spin textures, has inspired proposals for their application in nanodevices. Here, the authors use Lorentz transmission electron microscopy to demonstrate edge-mediated skyrmion chain formation in FeGe nanostripes.

## Graphical abstract

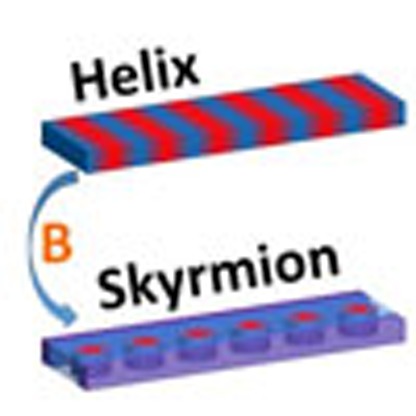

Since the discovery of magnetic skyrmions in 2009 (ref. 1), they have attracted a lot of attention in condensed matter physics because of their rich physics as well as their potential application in spintronic devices^2^,^3^,^4^. A skyrmion is a vortex-like spin texture, in which local magnetic moments point in a concerted manner effectively wrapping a sphere^5^,^6^. The peculiar twists of the spins within the skyrmion make it possess a nontrivial topology, which imparts an emergent electromagnetic field on electrons passing through it, yielding unconventional spin-electronic phenomena^7^ such as the ultra-low threshold current density for skyrmion motion^8^. This property, together with its tunable small size and topological stability, presents the skyrmion as a promising candidate for building the next generation's high-density but low-dissipation memory devices. A typical example is the skyrmion-based race-track memory, where the nanostripe is encoded via a precise control of a chain of individual skyrmions using spin polarized current pulses^9^,^10^,^11^. Recently, many theoretical studies have demonstrated the possibility of hosting and manipulating individual skyrmions in such confined geometry^12^,^13^,^14^. However, realization of individual skyrmion chains in the nanostripe has not been attained experimentally^15^.

The capacity to image the spin configurations in nanostructured helical magnets can provide valuable insight into this issue. High-resolution Lorentz transmission electron microscopy (TEM), which uses the Fresnel method (Supplementary Note 1 and Supplementary Figs 1 and 2) to obtain the nanometer-scale magnetic structures, has achieved great success in identifying skyrmion crystals in helical magnetic thin films^2^,^16^. In principle, it should be possible to adapt this technique to observe the magnetic state in ultra-narrow stripes. However, the sharp interface between sample and vacuum would lead to strong Fresnel fringes and give rise to artificial magnetic contrast around the edge. As a result, the real spin configurations around the edge are severely smeared out in such small-size samples^15^,^16^,^17^. Another challenge is the difficulty in obtaining nanostructured helical magnets with the desired geometries. Recent advances have enabled the fabrication of high-quality MnSi nanowires synthesized by chemical method, but the complex crystal structure in the nanowires, such as the parallelogram cross-section and twin boundaries, is a big challenge in obtaining the skyrmion chain under a magnetic field aligned perpendicular to the nanowire, although individual skyrmion cluster states can be identified in the magnetic fields along the long axis of the nanowire^18^,^19^.

Here, we fabricated FeGe nanostripes with well-defined geometries by a top–down method. By coating the amorphous PtCx at the edge of the nanostripe, we significantly reduced the interfacial Fresnel fringes in Lorentz TEM images. Eventually, a single skyrmion chain (SSC) was unambiguously observed in the nanostripe with its width comparable to the featured skyrmion size. Further systematic investigation of the magnetization dynamics of nanostripes with various widths unveils an edge-mediated mechanism to create skyrmions in confined geometries at low temperatures.

## Results

### The magnetization process in 130 nm wide FeGe nanostripe

We choose the B20 compound FeGe to demonstrate the effect of confined geometry on the formation and stability of skyrmions. FeGe is a typical helical magnet harbouring a skyrmion phase near room temperature. Bulk FeGe samples exhibit a paramagnet to helimagnet transition at a Curie temperature *T*_c_∼280 K under zero magnetic field^20^. The spin helix possesses a long range wavelength, *λ*∼70 nm, and a fixed wave-vector, **Q**, along the high symmetry crystal axis. If a finite magnetic field, **B**, is applied at the temperature *T*<*T*_c_, it becomes energetically favourable to form a conical phase with **Q**||**B**. At an even higher field, the conical phase transfers into ferromagnetic spin alignment. Both helical and conical states belong to single-twist magnetic structure as the rotation of their magnetization is only in one direction fixed by the wave-vector **Q**. In contrast, in a skyrmion phase, the magnetization rotates in two directions, forming double-twist modulations, which occupy only a tiny pocket in the *T*–*B* phase diagram slightly below *T*_c_ in bulk samples (Supplementary Fig. 3). This region of stabilizing skyrmion phase would be, to some extent, expanded in the *T*–*B* space when the sample becomes extremely thin^2^,^16^. The evolution of spin configurations with varied **B** and *T* in FeGe reflects a common behaviour in helical magnets^6^.

**Figure 1:**
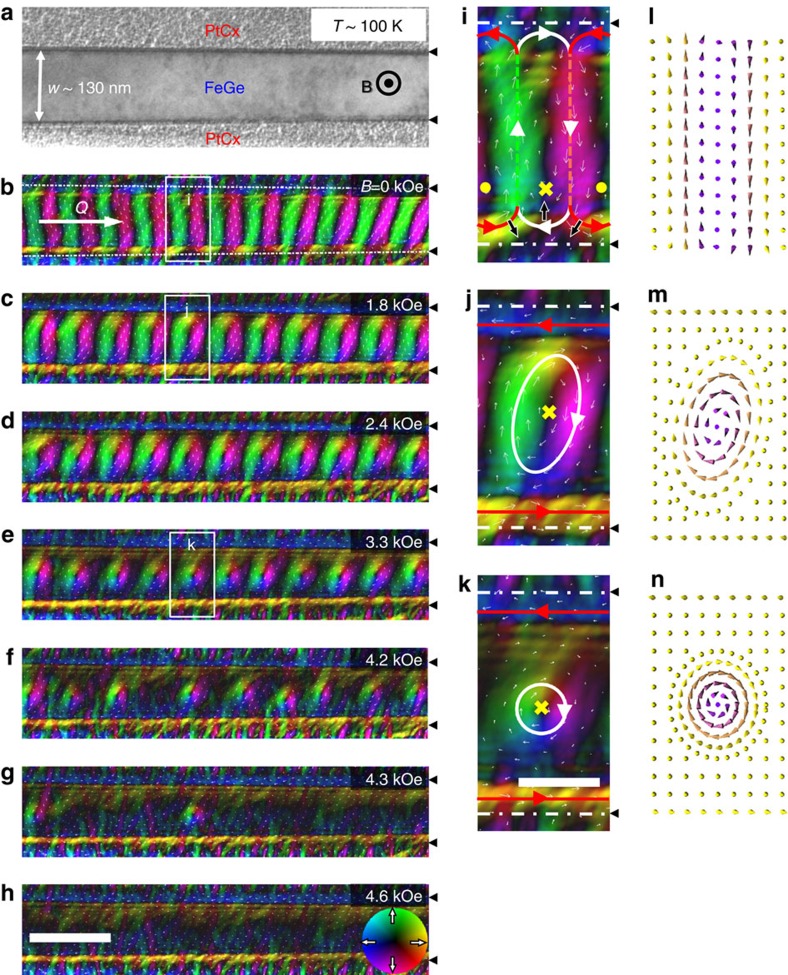
Variations of spin texture with magnetic field in a 130-nm FeGe nanostripe at the temperature *T*=100 K. (**a**) TEM image of the FeGe nanostripe surrounded by an amorphous PtCx layer. The magnetic field, **B**, is applied normal to the stripe plane. *w* stands for the width of the nanostripe. (**b**–**h**) Magnetic-field dependence of the spin texture, represented by the lateral magnetization distribution as obtained by transport-of-intensity equation (TIE) analysis of three Lorentz TEM images with the defocus values of **−**144 μm, 0, +144 μm. The colour wheel represents the magnetization direction at every point. ‘**Q**' is the wave vector. (**i**–**k**) Enlarged regions marked by the white boxes in corresponding panels (**b**–**e**). The small white arrows represent the in-plane magnetization direction at each point. · and × stand for the upward and downward directions of spins, respectively. At *B*∼0 Oe, spin helices are terminated at the edge and form two types of half-disk domains by distinguishing the curling direction of in-plane magnetization around the edge (thick red lines, anti-clockwise; thick white lines, clockwise). The magnetic field-driven evolution of half-disk domains is illustrated by using small black arrows to point out the moving directions of the domains in **i**. (**l**–**n**) The schematic spin arrangements are shown in the corresponding panels **i**–**k**. The white dot lines in **b** stands for the FeGe/PtCx interfaces, which are marked by small black triangular in the magnetic images **b**–**h** and **i**–**k**. The scale bars in **b**–**h** and **i**–**k** are 200 and 60 nm, respectively.

To study the effect of geometric confinement on the formation and stability of skyrmions, a series of FeGe nanostripes with various widths were fabricated from the bulk by a complex process (Supplementary Note 2 and Supplementary Fig. 4). Figure 1a shows a typical TEM image of the nanostripe with a width of *w*∼130 nm, which is comparable to the featured skyrmion lattice constant, *a*_sk_ (
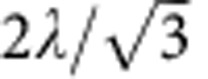
∼81 nm). The surrounding grey part is the amorphous PtCx layer, which can significantly reduce the effect of Fresnel fringes on the real magnetic structure around the edge and enable us to observe directly the magnetic structure in the ultra-narrow stripes (Supplementary Fig. 5). An external magnetic field, **B**, is applied perpendicular to the stripe plane with its direction pointing upward (marked as black ·). Figure 1b–h shows the evolution of the spin textures of this nanostripe with increasing *B* measured at 100 K, where the planar magnetic distributions were obtained by the magnetic transport-of-intensity equation (TIE) analyses^2^ of the high-resolution Lorentz TEM data (Supplementary Fig. 2). The two FeGe/PtCx interfaces, marked by the white dotted lines, would induce weak artificial magnetic contrast in the PtCx region because of the Fresnel fringes (Supplementary Note 3 and Supplementary Fig. 5). In the following, we will focus on the magnetic state in the FeGe parts by marking the boundary with a small black triangle. At low-magnetic field, a spin helix is observed with a period of 70 nm (Fig. 1b), consistent with the period in the bulk sample^16^. But, the wave-vector, **Q**, is almost parallel to the long axis of the nanostripe. With increasing *B*, the magnetization undergoes a series of dynamical processes (Fig. 1c,d), and eventually evolves to circular skyrmions at *B*∼3.3 kOe (Fig. 1e,k). These skyrmions assemble into a perfectly single chain because of the transverse constriction. This SSC in the interior of the nanostripe is accompanied by a chiral magnetization at the edge, as indicated by the red arrows in Fig. 1k. Spins on the two edges tilt onto the plane, and orient their planar components parallel to the boundary (schematic diagram in Fig. 1n). That is because of an unbalanced torque acting on the edge spin originating from the Dzyaloshinkii–Moriya interaction^10^,^11^,^21^. Reversal of the sample chirality would flip the edge spin orientations. Higher fields above *B*∼4.2 kOe melt the perfect SSC state into isolated skyrmions confined in the nanostripe (Fig. 1f,g), and finally polarize all the interior magnetizations at about *B*∼4.6 kOe (Fig. 1h), where magnetic contrast of the planar components disappears. However, the chiral edge magnetization is still persistent.

At elevated temperatures, the SSC still persists. The *T–B* phase diagram of the 130-nm wide nanostripe (Supplementary Fig. 6) explicitly shows a highly stable SSC in this confined geometry, where the SSC survives in the whole temperature region accessible by our Lorentz TEM experiments. This lowest temperature (∼100 K) is far below the magnetic transition temperature *T*_c_(∼280 K)^20^ and is even much lower than 200 K, the lower bound of the skyrmion phase reported in two-dimensional (2D) FeGe plates with the same thickness of *t*∼65 to the 130-nm nanostripe^16^. Very likely, the SSC phase extends through the whole low-temperature regime in this confined geometry. The comparable size between the nanostripe and single skyrmion allows us to identify that such a narrow stripe (*w*∼130 nm) can only accommodate one skyrmion in the transverse direction by simple geometry analyses. However, the wider extension of temperature for hosting the skyrmion phase in the nanostripe is in sharp contrast to the above-mentioned low stability of skyrmions in bulk^20^ or the thickness-dependent stability in two-dimensional films^16^. Obviously, this observation suggests an important role played by geometric confinement on the formation of skyrmions.

To explore the physical origin of this effect, we closely examined the magnetization process. Figure 1b shows the spin helix at *B*∼0 Oe. Unlike the locking of wave vector, **Q**, along the high-symmetry crystal axis because of the weak crystal anisotropy reported so far^6^, we noticed that the spin helix in narrow stripes always orients its wave vector parallel to the edge of the nanostripe. Such sample-shape-dependent orientation of stripe domains has been addressed in the perpendicular anisotropic magnet^22^,^23^, where lowering the stray field energy gives rise to perpendicular or parallel stripe domains at the edge of the sample, with all other orientations being less energetically favourable. In this case, spin helices are terminated at the edge, forming periodically modulated half-disk domains (the details on the edge state are depicted in Supplementary Note 4 and Supplementary Figs 7 and 8). Magnetic moments in these domains have fixed swirling directions (Fig. 1i,l), which are indicated by white (clockwise) and red (counter-clockwise) arrows, respectively. Moments at the disk centre are out of plane with staggered polarity upward (marked as ·) and downward (marked as **×**). Although the in-plane magnetization can be directly obtained by TIE analysis, the polarity is inferred by tracing the evolution of magnetization under high magnetic fields, by the fact that a large enough *B* aligns spins along the same direction. Owing to the chiral property of helical magnets, the rotation direction and polarity of disks are entangled so that only two types of half disk domains can occur. These two types of domains alternately appear along the edge as they are coupled to the periodically modulated helices inside the stripe.

When a weak external magnetic field is applied, the Zeeman energy enforces an increase in the proportion of the upward spins. According to the Lorentz TEM data, the increasing number of upward spins is accommodated by expanding the boundaries of the red half-disk domain (the moving directions are marked by small black arrows with white frame) as its polarity in the centre is parallel to the magnetic field. Above a threshold value of magnetic field *B*∼1.8 kOe, the edge spins coalesce in the red parts, forming a uniform edge state (red straight arrows in Fig. 1j and details in Supplementary Fig. 7)^11^,^12^,^21^, whereas the white parts are lifted away from the edge, forming half-skyrmions, that is, merons^24^,^25^. Another meron is similarly formed at the opposite edge, and together they constitute a bimeron (Fig. 1j,m), which can be regarded as an elongated skyrmion as it carries the same unit of topological charge as a skyrmion. With a further increase of magnetic field up to ∼3.3 kOe, the elongated skyrmions are compressed (Fig. 1k,n), and form a perfect SSC with the topological charge conserved.

### The magnetization process in 396 nm FeGe nanostripe

**Figure 2:**
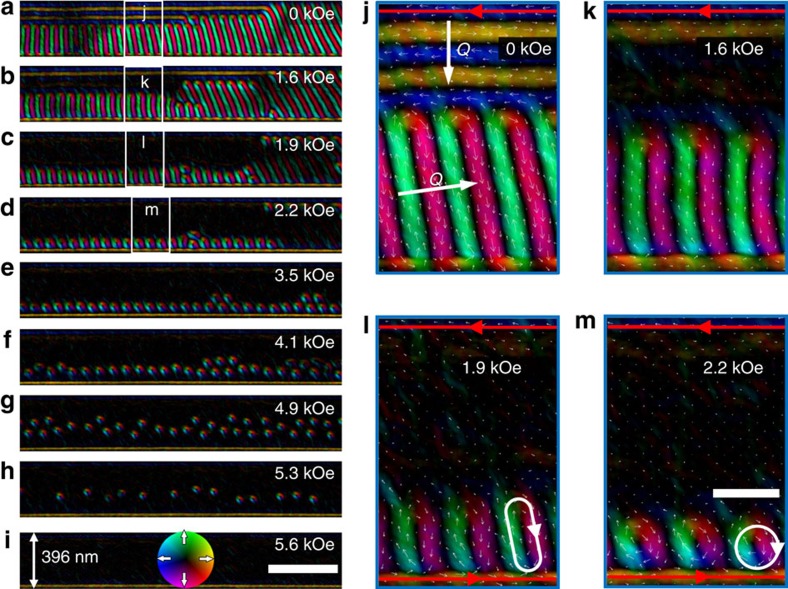
Variations of spin texture with magnetic field in a 396-nm FeGe nanostripe at *T*=100 K. (**a**–**d**) The different behaviour of spin helices with wave vectors **Q ||** edge and **Q ⊥** edge in a magnetic field. (**j**–**m**) The corresponding enlarged region marked by the white boxes in the corresponding panels (**a**–**d**). Under the applied magnetic field, the spin helix with almost **Q ||** edge transfers into skyrmions, whereas those with almost **Q ⊥** edge disappear directly without leaving skyrmions. (**e**–**g**) The collective movement of a skyrmion chain into the interior of the stripe with increasing magnetic field. (**h**–**i**) The transition of the isolated skyrmions to ferromagnetic spin textures with uniform edge state. The red arrows indicate the orientation of the magnetization around the edge. To show magnetic structure in FeGe nanostripe more clearly, all the images are tailored to remove the PtCx part. The defocus values used for Lorentz TEM imaging are −192 μm, 0, +192 μm. The scale bars in **a**–**i** and **j**–**m** are 500 and 100 nm, respectively.

The magnetization process in the 130-nm-wide nanostripe provides a strong hint that the creation of skyrmions in confined geometries is closely related to the presence of half-disk domains, originating from spin helices propagating along the edge. To test this idea, we systematically investigated the magnetization dynamics of nanostripes with increasing width while fixing the low temperature *T*∼100 K. Figure 2a–i shows a representative evolution of spin textures in a *w*∼396 nm nanostripe. The corresponding enlarged images are selectively illustrated in Fig. 2j–m. Compared with the *w*∼130 nm stripe, the 396-nm stripe is nearly five times larger than the skyrmion lattice constant ∼81 nm, and shows a helical state with the co-existence of orthogonal wave vectors **Q ||** edge and **Q ⊥** edge (Fig. 2a,j). Similar to the *w*∼130 nm nanostripe, the bottom spin helices with **Q ||** edge possess a distorted edge state. In contrast, the upper spin helices with **Q ||** edge, being parallel to a uniform edge state, exhibit a perfect helical phase without distortion.

When the magnetic field is turned on, the two orthogonal helices show remarkable differences. At *B*∼1.6 kOe (Fig. 2b,k), the helix with **Q ⊥** edge becomes less visible, whereas the helix with **Q ||** edge begins to deform in the same way as the *w*∼130 nm nanostripe. When the magnetic field is further increased to ∼1.8 kOe, the helix with **Q ⊥** edge disappears, whereas the other with **Q ||** edge gives rise to compressed bimerons (Fig. 2l), which eventually self-organize to skyrmion chains positioned at the edge at elevated magnetic field (Fig. 2d,m). This observation indicates that skyrmions can only be created from helices with a distorted edge state. This scenario works well at low temperatures where the magnetization dynamics are more important than the statistics. In the process, the high mobility of skyrmions is also illustrated in the confined geometry, where the chain is easy to move under the action of the magnetic field. This leads to the unfixed positions of skyrmions. For example, a skyrmion chain around the upper right edge at *B*∼2.2 kOe in Fig. 2d merges into the bottom skyrmion chain at *B*∼3.5 kOe (Fig. 2e) without any creation or annihilation of skyrmions. Similar phenomena are also found in a 306-nm-wide stripe with certain defects (Supplementary Fig. 9) and 550-, 1,017-nm-wide stripes (Supplementary Fig. 10). In spite of this high mobility, there is almost a one-to-one correspondence between the number of these helix periods and the number of skyrmions.

Once the skyrmion chain is formed at the edge (Fig. 2d), another interesting observation is that the skyrmion chain tends to move collectively into the interior of the stripe (Fig. 2d–g) with increasing magnetic field, and the number of skyrmions remains unchanged over a wide interval of magnetic fields (2.2 kOe<*B<*4.9 kOe). The fixed number of skyrmions supports the topological stability of skyrmions that they become robust once skyrmions are created^5^. The collective movement of skyrmions can be readily explained by the repulsion between edge spins and skyrmions^26^, conversely confirming the well-known edge state. Consequently, a distorted skyrmion chain (DSC) far away from the nanostripe (Fig. 2g) is formed. Finally, at even higher magnetic fields with *B**>***5.3 kOe, the topologically stable DSC melts and skyrmions therein gradually disappear, as a transition to the ferromagnetic spin texture occurs (Fig. 2h,i).

This self-organized skyrmion chain at the edge induced by the helices with the distorted edge state and the subsequent collective movements under the action of magnetic fields are found to be a universal phenomenon that is extensively verified at low temperatures in nanostripes of various widths (Supplementary Figs 9 and 10). Moreover, as the lengths of nanostripes are finite in our experiments, the skyrmion chain is also observed at the end edge of the wide nanostripes as long as the helices with distorted edge state exist. These experimental results all point to the conclusion that the spin helices with distorted edge state underlie the emergence of skyrmion chains. In other words, we have identified an edge-assisted mechanism to create skyrmion chains in nanostripes instead of the previously reported skyrmion crystal, where thermodynamics plays the key role^2^,^16^. This self-organized chain in our system is completely distinct from the previously reported preferential formation of skyrmions near the sample edge in 2D plates of FeGe^16^. Those samples, fabricated by traditional argon-ion thinning methods, have an inhomogeneous thickness with the thicker part in the interior and the thinner part at the edges, where skyrmions around the edge are mainly stabilized by the reduced thickness.

### Temperature dependence of skyrmion arrangement

**Figure 3:**
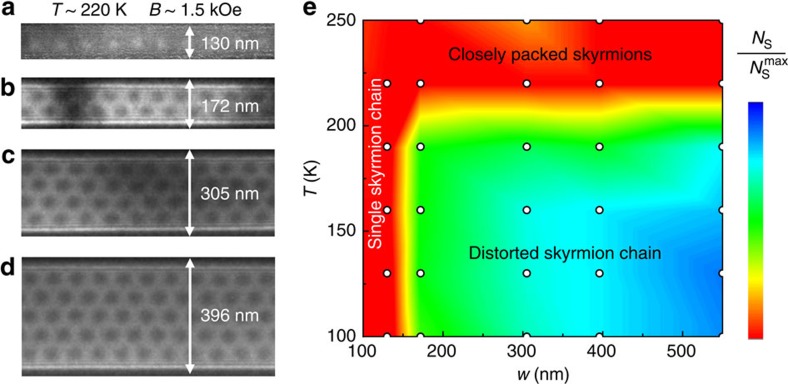
Sample width dependence of the skyrmion arrangement in the temperature (*T*)-width (*w*) diagram. (**a**–**d**) The width dependence of closely packed skyrmion arrangements obtained at an elevated temperature, *T*=220 K as a magnetic field was applied. The image is acquired under over-focused condition with the defocus value 288 μm. The dark or bright circles represent the skyrmions. The change of dark or bright is due to the reversal of the crystal chirality. (**e**) Sample width dependence of the skyrmion phase diagram in the plane of temperature and width. The open dots are data points from the Lorentz TEM measurement. From these data, a coloured map is constructed to show the normalized skyrmion density, defined as 
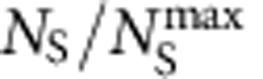
, with *N*_S_ the actual number of skyrmions at each temperature, and 
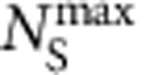
 the maximum number of skyrmions that can be accommodated in the nanostripe.

To gain a more thorough understanding of skyrmion formation in confined geometries, elevated temperatures were also investigated (Fig. 3a–d), where thermal fluctuations would be dominant compared with the magnetization dynamics. It is well understood that a closely packed skyrmion (CPS) crystal is favoured by thermal fluctuations in bulk or 2D films. This crystallization of skyrmions is ascribed to inter-skyrmion interactions^6^, which is an intrinsic property of skyrmions and should be independent of material details and sample sizes. Therefore, the CPS structure is expected in nanostripes when the temperature is high enough. This prediction is clearly supported by Lorentz TEM images of nanostripes, which show that the skyrmions are closely packed (Fig. 3a–d and Supplementary Fig. 11) with the number of skyrmions in the transverse direction proportional to the width of nanostripes.

Based on all the Lorentz TEM data on nanostripes with various widths measured at different temperatures, we constructed a full *T*-*w* phase diagram of the skyrmions in nanostripes (Fig. 3e). Although the maximum number, 
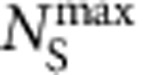
, of skyrmions in CPS that can be accommodated into the defined nanostripe is fixed, the actual skyrmion number, *N*_S_, however, depends on the temperature and the applied magnetic field. Here we define *N*_S_ at each temperature from the point at which the skyrmion number in the SSC or DSC starts to decrease under further increase of the magnetic field *B*. In other words, *N*_S_ is the actual maximum skyrmion number at a defined *T* under the application of a moderate magnetic field. Clearly, the CPS appears only at high temperatures. At temperatures below about *T*<200 K, skyrmions in the nanostripe appear in the form of SSC (when *w*<160 nm) or DSC (when *w*>160 nm). These results reveal the importance of magnetization dynamics in skyrmion formation at low temperatures.

## Discussion

We have demonstrated that the edge can be used to control skyrmion formation in confined geometries. At high temperatures, skyrmions tend to form a closely packed structure. This skyrmion arrangement is consistent with previous experimental results on the skyrmion crystal in the corresponding 3D or 2D FeGe compounds^1^,^2^. In this case, a SSC can be obtained by reducing the width of the nanostripe to the featured skyrmion size. More importantly, we show that the SSC persists even in the much wider nanostripes at low temperatures. We further identified that the skyrmions originate from the sample-shape-dependent orientation of the helical state. The helix with distorted edge state evolves into skyrmions, whereas those without distorted edge state transfer into ferromagnetic state directly. Taking the topological stability of skyrmions into account, it is commonly concluded that skyrmion cannot easily be destroyed and created from the single-twist magnetic structure including helical and conical phase. By contrast, the confined effect gives rise to the distorted edges states, which show half-disk arrangements, indicating their multi-twist modulations. Our results thus indicate the creation of the skyrmion from the multi-twist magnetic state is much easier than that from single-twist magnetic state. These findings offer a fundamental insight into the dynamical mechanism of the formation and evolution of skyrmions in a confined geometry, and might improve the viability of proposals for using skyrmions in magnetic nanostripes as the carriers of high-density information through the edge/or defect-engineering.

In addition, we have further observed the collective motion of the skyrmion chain, accompanied by a series of adjustment in the chain space and position. This motion is the direct result of skyrmion–skyrmion and skyrmion–edge repulsions. Presence of spins distortion around the edge is a fundamental property in geometry-confined nanostructures. This edge-mediated mechanism together with control of temperature and thickness could provide a powerful approach for tailoring the desired skyrmion properties in confined geometries.

## Methods

### Bulk sample preparation

The polycrystalline B20-type FeGe samples were synthesized with a cubic anvil-type high-pressure apparatus. A mixture of the elemental materials with an atomic ratio of 1:1 was synthesized by electromagnetic induction melting in an argon atmosphere. The compound was placed into a cylindrical BN capsule and was heat treated for 1 h at 1,073 K under a high pressure of 4 GPa. Structural characterization by X-ray diffraction and susceptibility measurements were consistent with the well-established structure and properties of bulk FeGe.

### Fabrication and characterization of the FeGe nanostripe

The nanostripes for TEM observation were prepared by lift-out method using a focused ion beam and scanning electron microscope dual beam system (Helios Nanolab. 600i, FEI) combined with a gas injection system and micromanipulator (Omniprobe 200+, Oxford). The details of the sample fabrication processes and characterization are shown in Supplementary Note 2 and Supplementary Fig. 4. The parameters of fabricated samples with varied width from 130 to 1,017 nm are summarized in Supplementary Table 1. In the main text, we mainly show the data from NS1 (*w*∼130 nm) and NS4 (*w*∼396 nm). Other data including some typical magnetization processes and the *T-B* magnetic phase diagram are shown in the Supplementary Information.

### Lorentz TEM measurements

We carried out the magnetic contrast observation by using the Lorentz TEM (JEOL-2100F). In Lorentz TEM mode, the objective lens was turned off and an objective mini-lens under the specimen was employed to induce the normal magnetic field to the thin nanostripe. The observed magnetic contrast can be understood qualitatively in terms of the Lorentz force acting on the moving electrons as they travel through in the magnetic foil. The high-resolution lateral magnetization distribution map was obtained by TIE analyses of the Lorentz TEM images. The details of the Lorentz TEM and the TIE analyses are given in the Supplementary Note 1. The magnetization distribution maps used in the main text and Supplementary Information are all original ones without subtracting the artificial magnetic contrasts. Thin-plate thickness was measured using Electron Energy Loss Spectroscopy (EELS). A double-tilt liquid-nitrogen cooling holder (Gatan, Cryo-Transfer Holder) was used to detect the phase transition below the Curie transition temperature *T*_c_. This enables the specimen temperature to be reduced to 100 K with the measured temperature displayed on the cooling holder controller. The magnetic field applied normal to the thin plate was induced by the magnetic objective lens of the TEM.

## Additional information

**How to cite this article:** Du, H. *et al*. Edge-mediated skyrmion chain and its collective dynamics in a confined geometry. *Nat. Commun.* 6:8504 doi: 10.1038/ncomms9504 (2015).

## Supplementary Material

Supplementary Information Supplementary Figures 1-11, Supplementary Table 1, Supplementary Notes 1-4 and Supplementary References
