# Supplementary material for: Edge-mediated skyrmion chain and its collective dynamics in a confined geometry
Source: Nat Commun. 2015 Oct 8;6:8504. doi: 10.1038/ncomms9504 (PMC4633724; doi:10.1038/ncomms9504)
Supplement: Supplementary Information — Supplementary Figures 1-11, Supplementary Table 1, Supplementary Notes 1-4 and Supplementary References [file ncomms9504-s1.pdf]

Supplementary Information

# Supplementary Figures

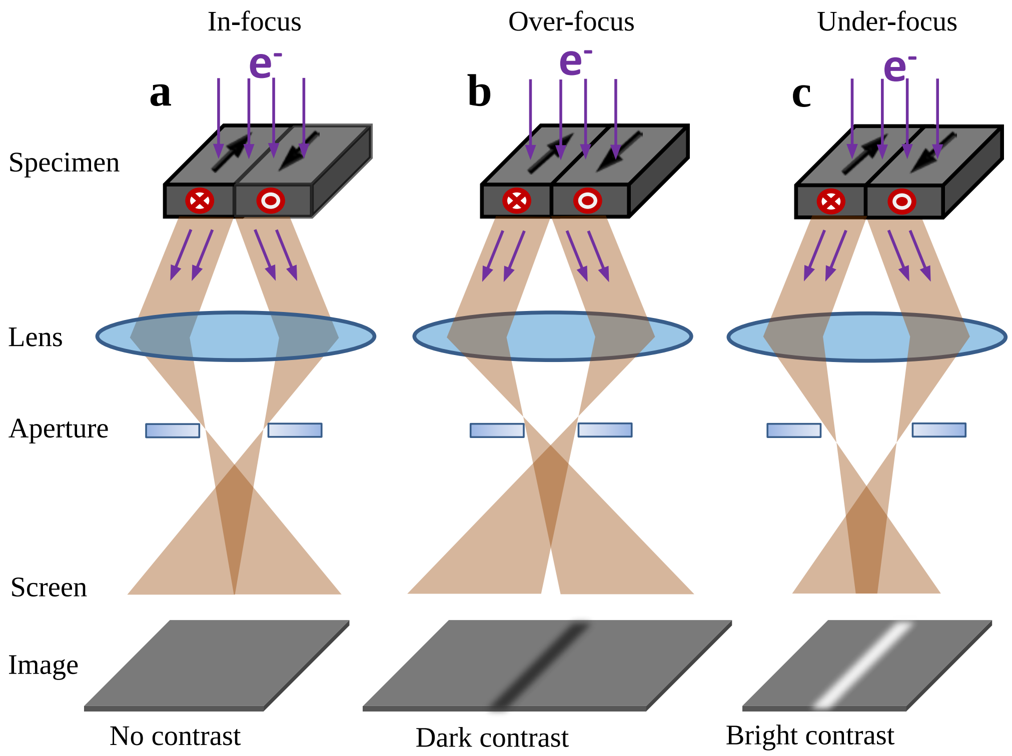


**Supplementary Figure 1 | The schematic ray diagram in a Fresnel image of a ferromagnetic specimen containing two 180º domain walls. (a),** in-focus conditions. The deflected electron beams are focused in the final image plane so that no magnetic contrast appears. **(b)** and **(c),** over- and under-defocus conditions. The deflected electron beams lead to dark and bright contrast, respectively.

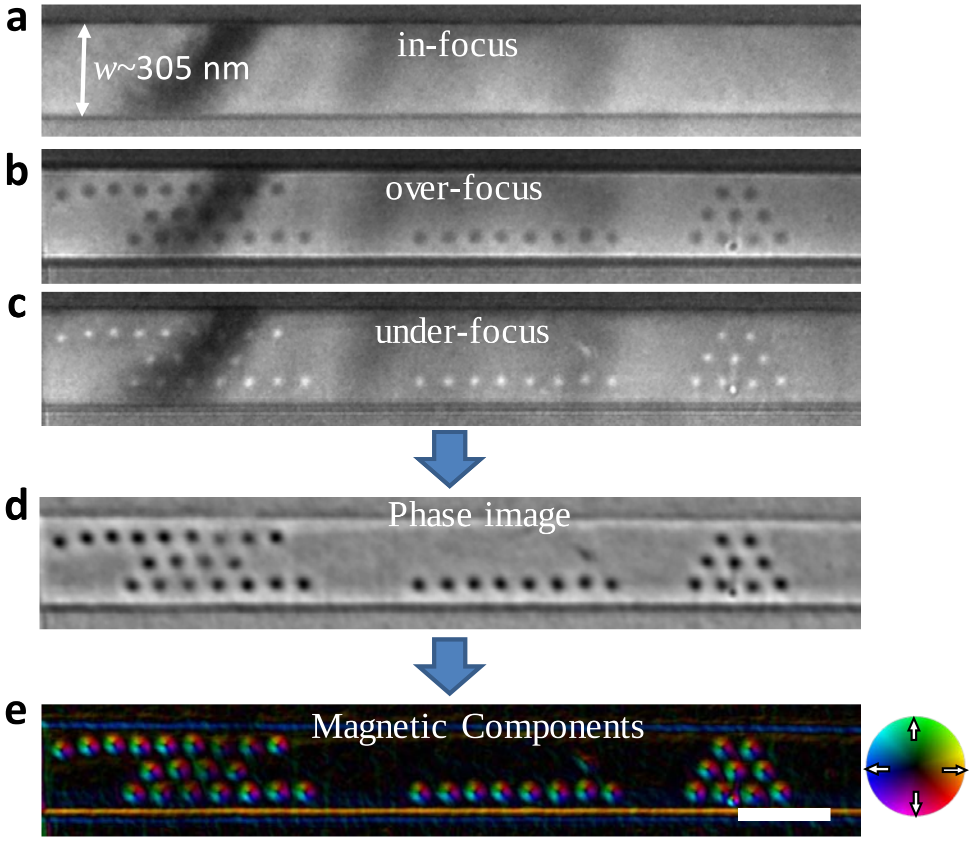


**Supplementary Figure 2 | Schematic diagram of magnetic TIE analysis in the skyrmion state.** Three images in the same region under different defocus conditions (**a**, in-focus; **b**, over-focus; **c**, under-focus; $\Delta z$ = 196 μm) are acquired by using Lorentz TEM. **(d)**, the phase image, which is created from the three Lorentz micrographs applying Eq. (1) in the Supplementary Note 1. **(e)**, the in-plane magnetization distribution map, which is generated from phase image by applying Eq. (2) in the Supplementary Note 1. The sample is named as NS3 with its detailed description in Supplementary Table 1 and Supplementary Figures 4 and 5. The color wheel represents the magnetization direction at every point. This color wheel is used in the whole text including the main part and Supplementary Information. Scale bar, 300 nm.

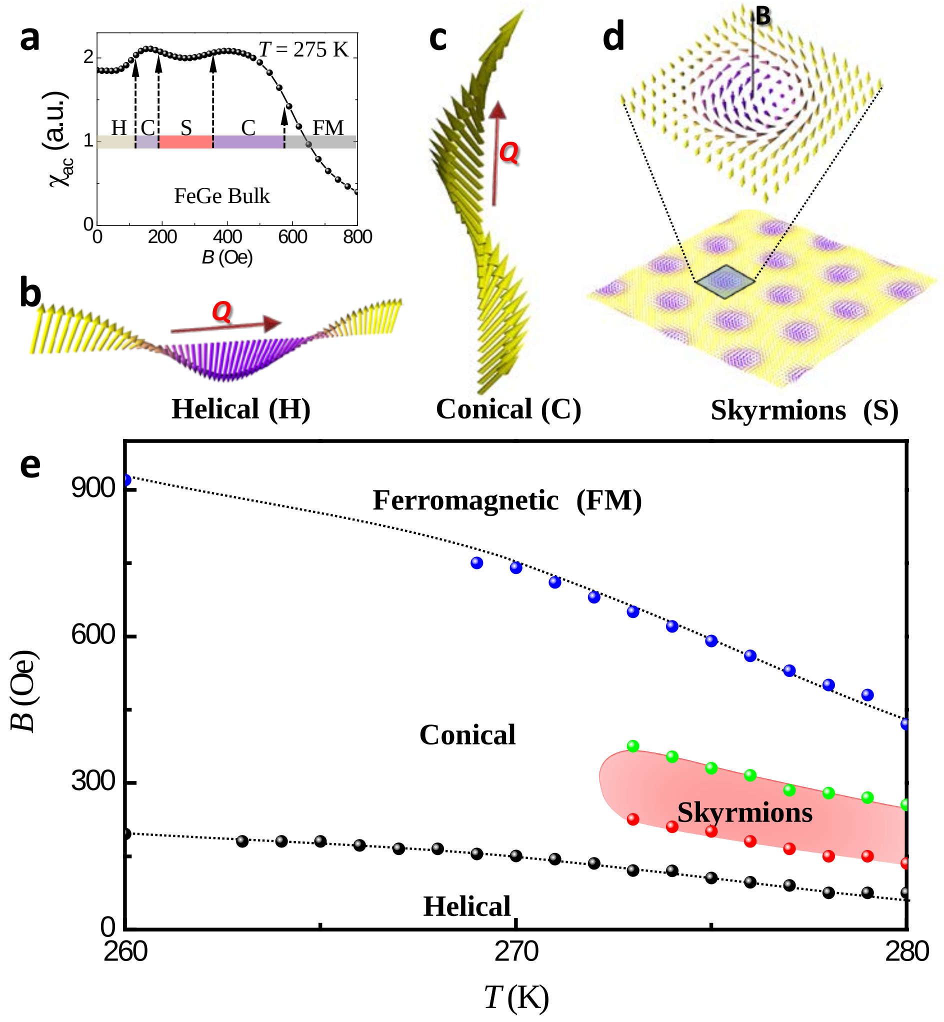


**Supplementary Figure 3 | Magnetic phase diagram of bulk FeGe inferred from the ac susceptibility. (a),** Typical isothermal ac susceptibility data at $T$ ~ 275 K. Subtle changes in $\chi_{ac}$ ($\boldsymbol{B}$) below 800 Oe indicate rich magnetic phases, which are identified by comparing the $\chi_{ac}$ ($\boldsymbol{B}$) curve with their characteristic forms in helical magnets [1-2]. **(b)**, **(c)**, and **(d)**, schematically, represent the spin configurations of helical, conical and skyrmion phases, respectively, which develop below $T_c$ ~ 280 K with the increase of magnetic field. Clearly, the helical order unpins under application of a high magnetic field to form the conical phase with its wave vector along the direction of magnetic field, and then condenses into skyrmion crystal with a hexagonal arrangement. **(e)**, phase diagram of magnetic structure in bulk FeGe inferred from the ac susceptibility. The ac susceptibility was measured on a physical property measurement system (Quantum Design, Inc.) with the excitation amplitude of 3.5 Oe and excitation frequency of 333 Hz.

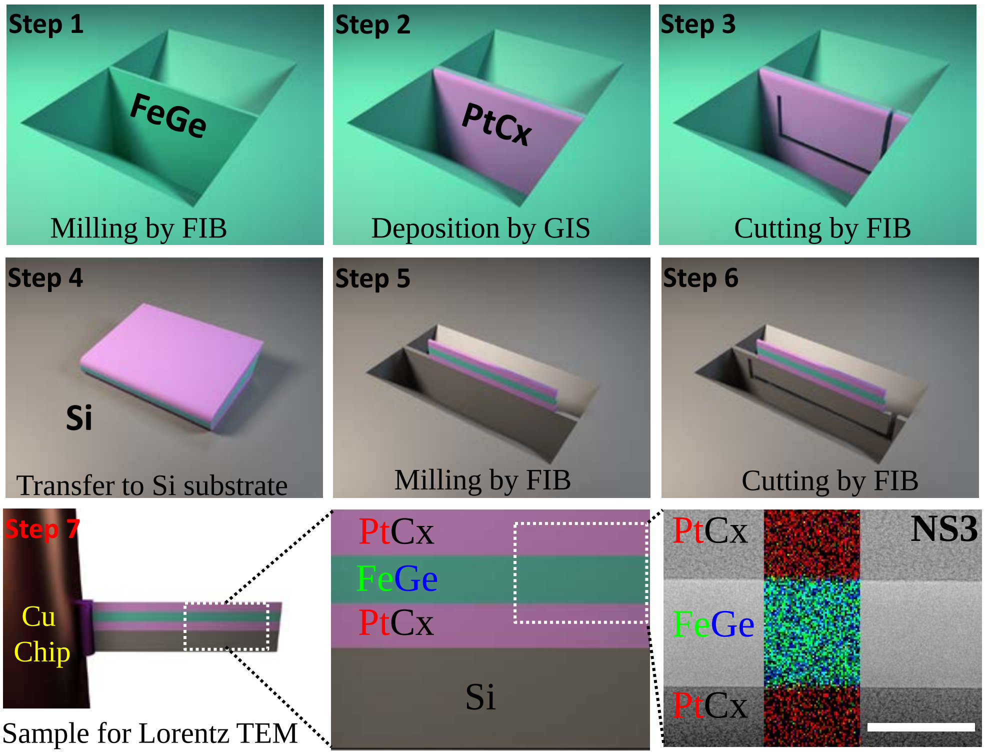


**Supplementary Figure 4 | Schematic procedure for fabricating the nanostripes by using focused ion beam and scanning electron microscopy (FIB-SEM) duel beam system (Helios Nanolab. 600i, FEI) equipped with a Gas Injection System (GIS), and Omniprobe 200+ micro-manipulator.** The whole process is schematically depicted from step 1 to 7. A typical nanostripe is shown in the final panel. The compositions are measured by energy dispersive spectroscopy (EDS, Oxford X-MaxN 80T) under scanning TEM (STEM) model with the operating voltage 200 kV. The compositional maps for Fe, Ge, and Pt with color superposition (Fe blue; Ge Green; Pt; red) show the clear FeGe/PtCx interface. The coating layer is the Pt nanocrystal with a typical size of 3-5 nm embedded into amorphous carbon matrix [3]. Since carbon is light element and the operating voltage in STEM mapping is high, the carbon is hardly resolved under this condition. Scale bar, 300 nm.

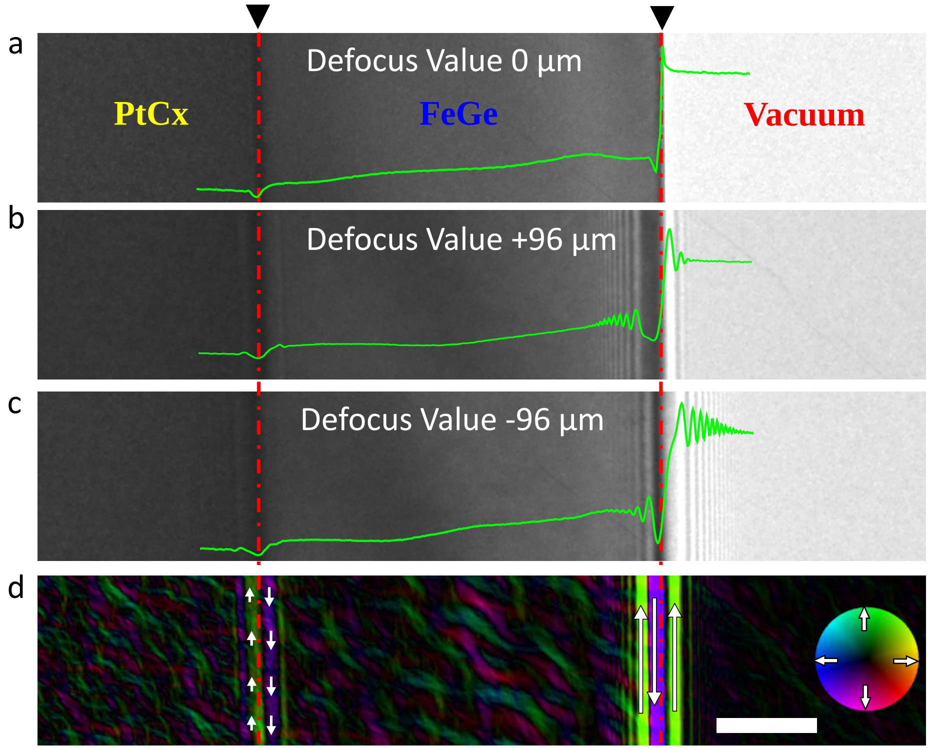


**Supplementary Figure 5 | Comparison of Fresnel fringes at PtCx/FeGe and FeGe/vacuum interfaces.** (**a**), under-focus case with the defocus value 96 µm; (**b**), in-focus case; (**c**), over-focus case with the same defocus value 96 µm. It is clearly shown that the range of Fresnel fringes extends above ~100 nm at the FeGe/vacuum interface in the under-focus or over-focus conditions, while it is below ~50 nm at the PtCx/FeGe interface (**a** and **c**). Moreover, compared with the FeGe/vacuum interface, the strength of Fresnel fringes at the FeGe/PtCx interface is also significantly weakened. This advance enables the Lorentz TEM to directly image the edge state with less influence by the Fresnel fringes. (**d**), the artificial in-plane magnetic components constructed by the TIE analysis based on the image of **(a-c)**. The significantly reduced Fresnel effect at the FeGe/PtCx interface is clearly observed. The red dot lines indicate the position of the FeGe/PtCx and FeGe/Vacuum interface. For clarity, the corresponding strength of Lorentz TEM images under different focus conditions is plotted as the green blue lines. The white arrows in **(d)** represent the direction of lateral magnetization. Scale bar, 100 nm.

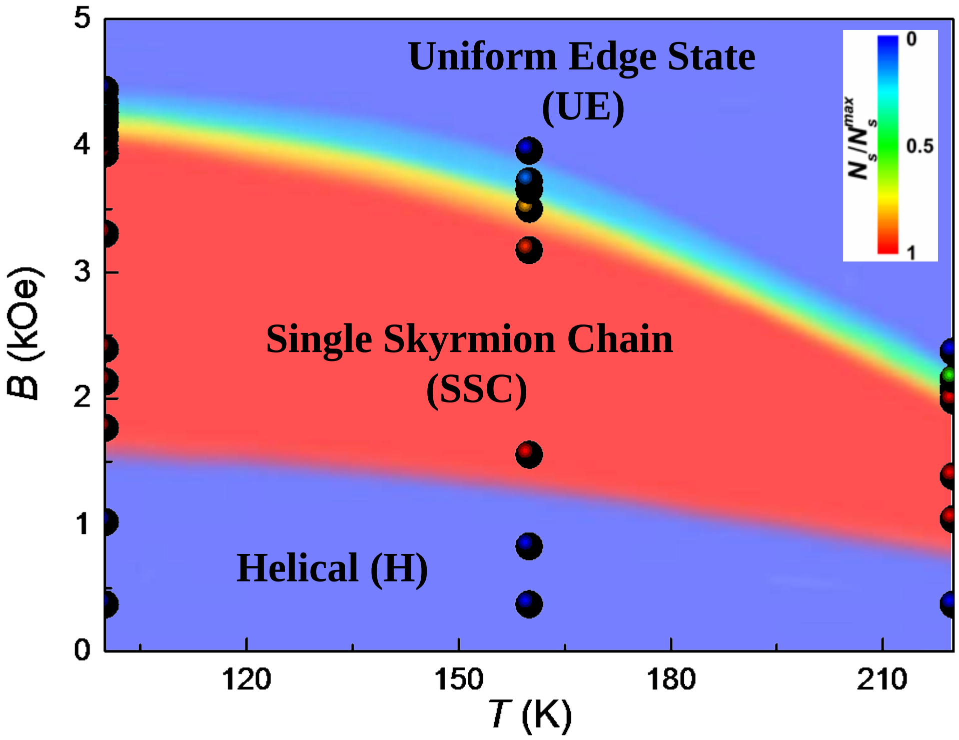


**Supplementary Figure 6 | Magnetic phase diagram of the 130 nm nanostripe in the *T-B* plane.** SSC represents the single skyrmion chain. The colored dots show the experimental points obtained by mapping of the normalized skyrmion density, from which the region of hosting SSC is marked in red for reference.

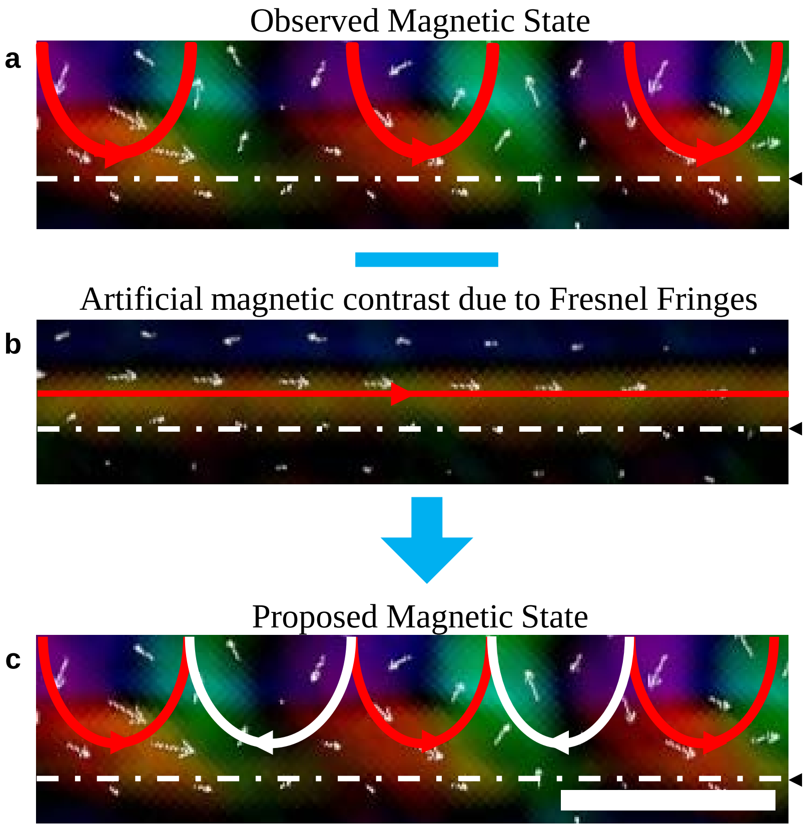


**Supplementary Figure 7 | Magnetic structure around the edge of a 305 nm nanostripe at zero magnetic field. (a)**, the observed spins arrangements. The red and white arrows stand for the counter-clockwise and clockwise half domains, respectively. **(b)**, the artificial spins arrangements due to the Fresnel fringes. The red arrows stand for the direction of the artificial magnetic moments. **(c)**, the proposed spins arrangements with the degenerate half domains. The white dashed-dot lines indicate the position of the FeGe/PtCx interface. Scale bar, 50 nm.

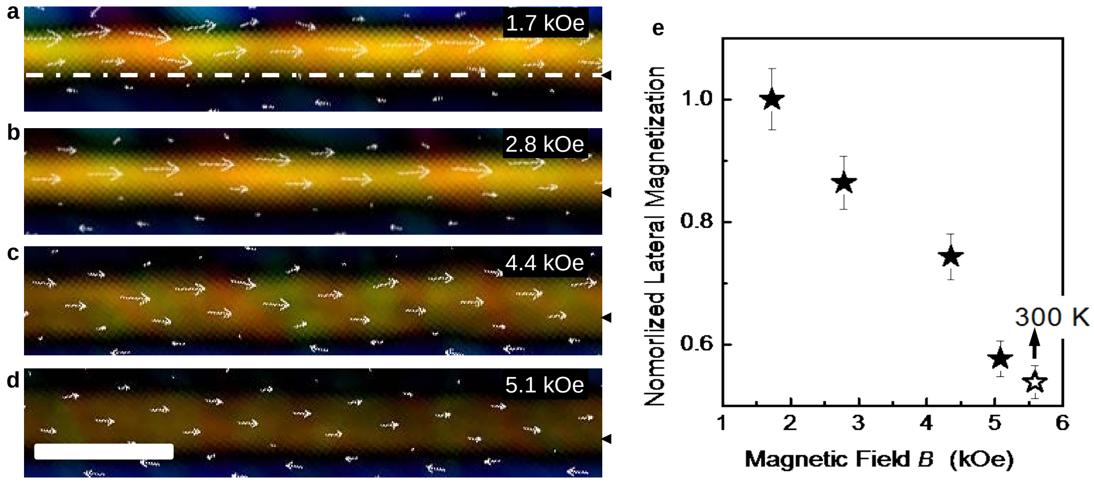


**Supplementary Figure 8 | Magnetic field dependence of reduced lateral magnetization at the edge of a 305 nm nanostripe. (a-d),** spin arrangements at the edge with varied magnetic field obtained at $T$ ~ 100 K. **(e),** the reduced magnetization as a function of the magnetic field. For comparison, the artificial magnetization is also plotted by the open star. The error bars are estimated by measuring the arrows length in the whole edge. Scale bar, 50nm.

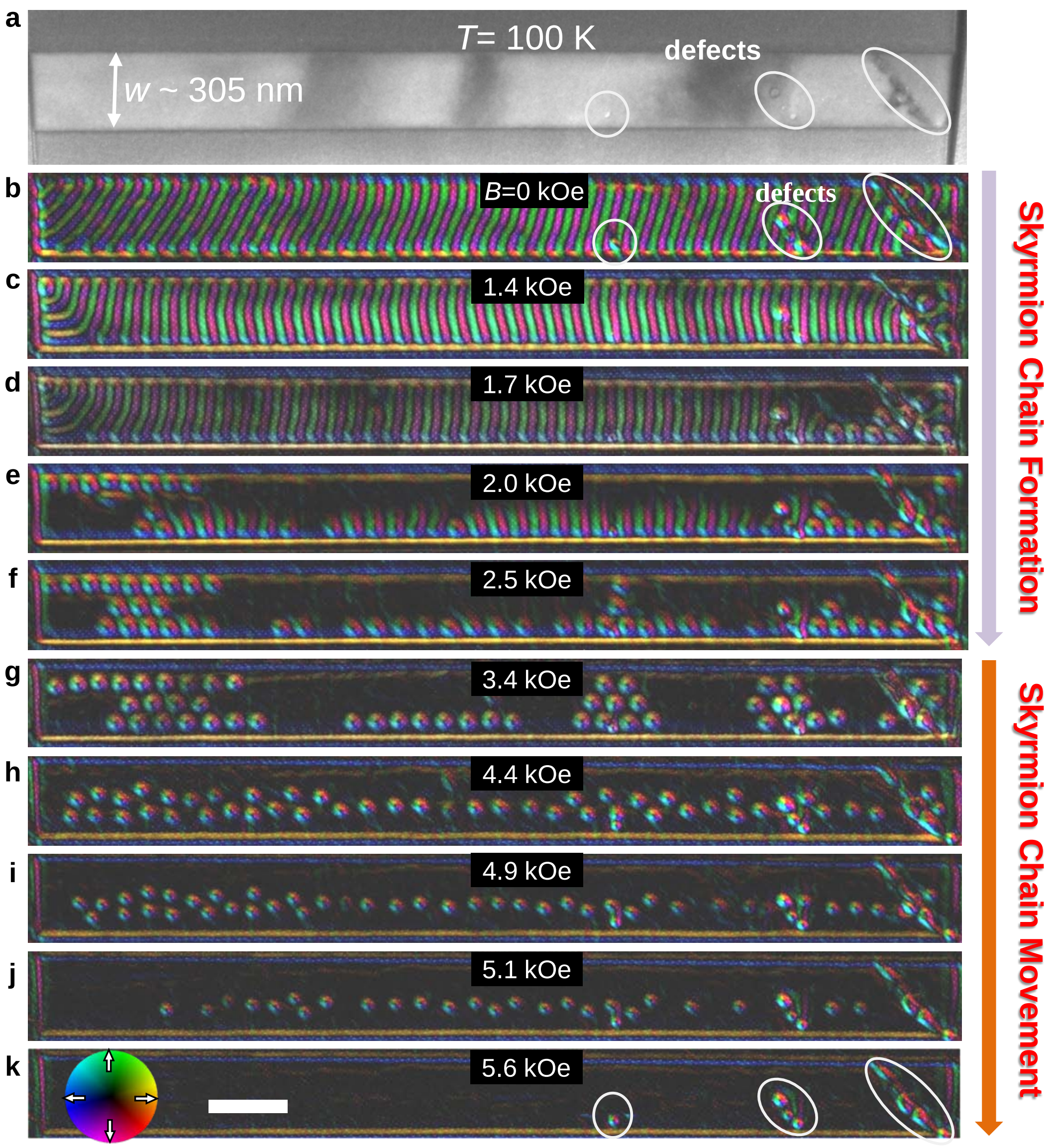


**Supplementary Figure 9 | The magnetization processes of a 305 nm FeGe nanostripe. a,** TEM image of the nanostripe. **(b-k)**, magnetic-field dependence of the spin textures obtained at 100 K. The white circles highlight the defects in the sample. At ***B*** ~ 0 Oe, the spins show a helical ground state with **Q** ǁ edge, the same to 130 nm stripe. When the magnetic field is switched on, the nanostripe shows essentially similar dynamics of magnetization with 130 nm or 396 nm nanostripes presented in the main text. Specifically, the self-organized skyrmion chains sit at the edges at low magnetic field, then decouple from the distorted edge state and move to the inner region of the nanostripe with increasing magnetic field. Defects including corner, dot or grain boundaries are also beneficial for the creation of skyrmions [4-5]. At low magnetic fields, skyrmions gather together around the defects to form clusters (**f** and **g**), but the total number of skyrmions in the stripe remains unchanged. Scale bar, 300nm.

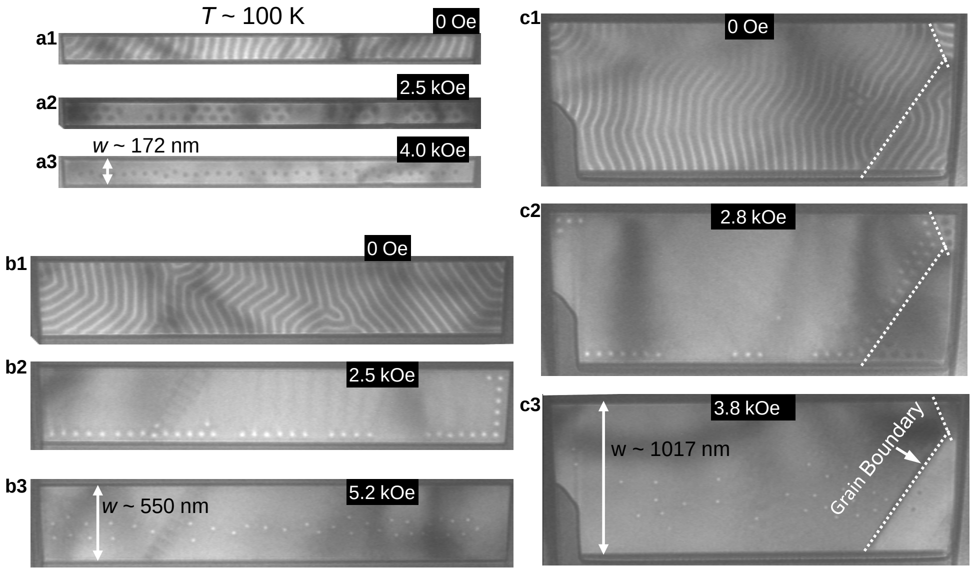


**Supplementary Figure 10 | The three typical magnetic structures (helical state, skyrmions around the edge, distorted skyrmion chain) of FeGe nanostripes with three widths at *T*~ 100 K under the applied magnetic field. (a),** *w* ~ 172 nm. **(b),** *w* ~ 550 nm. **(c),** ~ 1017 nm. All samples follow similar magnetization dynamics as shown in Fig. 2 in the main text that the helical states with distorted edge spins evolve into the skyrmions, which assemble in the form of chains around the edge at low field and move collectively into the center of the nanostripes at high field. This observation indicates that the mechanism of the edge-mediated skyrmion chain is independent of the sample width. Notice that the grain boundaries may also play a similar role to the edge in creating skyrmions at low temperatures, as shown in the 1017 nm nanostripe. It is worthwhile mentioning that the skyrmion chain or helical state is able to move or oscillates under the action of the magnetic field, especially in wide samples. The high mobility of skyrmions leads to deviations of the skyrmion relative to its initial positions. But, there is nearly a one-to-one relationship between the number of created skyrmions and the completed edge distortion. In particular, for the polycrystalline 1017 nm sample, two small regions divided by grain boundaries meet this one-to-one correspondence too. In the wider samples, the skyrmion chain preferentially forms along the bottom edge of the nanostripe, which is probably due to the slightly inhomogeneous thickness of the nanostripes. In a narrow stripe *w* ~ 172 nm, skyrmions formed on both sides. We have also fabricated samples with larger size, but the increased defects such as the grain boundaries and the inhomogeneous thickness make the situation more complicated. A detailed discussion of these results is beyond the scope of this paper. The images are acquired under over-focus conditions with the defocus value 192 µm. The dot lines indicate the grain boundary. The skyrmions are displayed as black or white circle dots. The change of magnetic contrast comes from the reversal of the chirality of the samples.

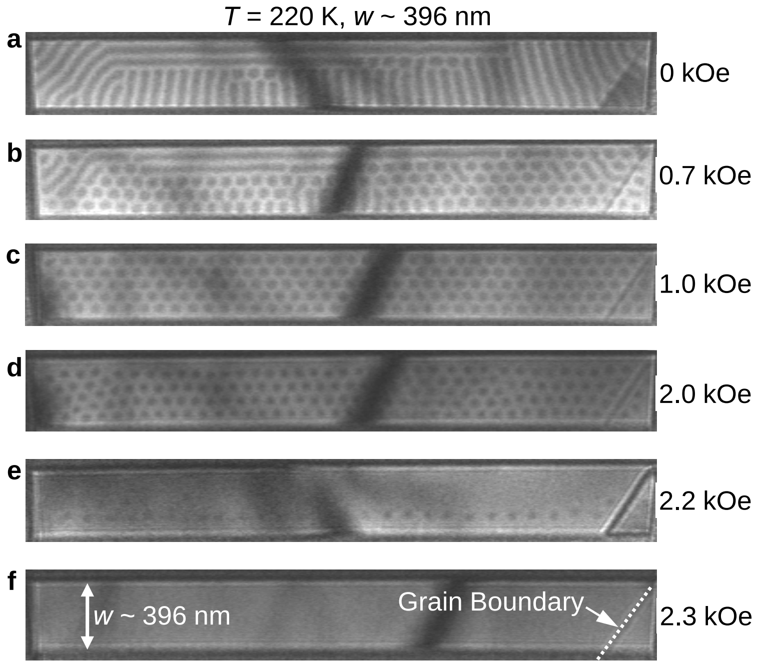


**Supplementary Figure 11 | The magnetic field dependence of spin textures in the 396 nm FeGe nanostripe at high temperature *T* ~ 220 K. (a)**, the helical state; **(b)**, mixture state of skyrmion lattice and helical phase; **(c)** and **(d)**, a packed skyrmion lattice; **(e)**, isolated skyrmions; **(f)**, a field-polarized ferromagnetic state surrounded by edge vortex state. The images are acquired under over-focus conditions with the defocus value 288 μm. The dot lines indicate the grain boundary.

# Supplementary Table

**Supplementary Table 1 | Parameters of FeGe nanostripes for Lorentz TEM imaging**

| Samples Name | Width(nm) (*w*) | Crystal plane | Thickness (nm) (*t*) |
|---|---|---|---|
| NS1 | 130 | [-123] deviation 5° | 65 |
| NS2 | 172 | [111] deviation 7° | 85 |
| NS3 | 305 | [111] deviation 7° | 82 |
| NS4 | 396 | [111] deviation 7° | 91 |
| NS5 | 550 | [112] deviation 15° | 92 |
| NS6 | 1017 | Polycrystalline | 90 |

# Supplementary Notes

## Supplementary Note 1. Lorentz TEM and magnetic transport-of-intensity equation (TIE) analyses.

The principles of Lorentz TEM can be understood classically in terms of the basic interaction between the electron beam and the magnetic fields within and around the magnetic specimen [6]. The most commonly used technique for revealing the domain structures is the Fresnel (or defocus) method [4-5]. In the method, the objective lens is defocused so that an out-of-focus image of the specimen is formed. The schematic ray diagram in the Fresnel model of the TEM is shown in the supplementary Figure 1. For the purpose of illustration, a simple specimen comprising two domains separated by 180$^{o}$ domain walls is assumed.

When the parallel electron beam passes through the area around the domain wall in the thin sample, Lorentz force, defined as $\mathbf{F} = e(\mathbf{v} \times \mathbf{B})$ with the electron velocity $\mathbf{v}$ and the magnetic induction $\mathbf{B}$, will lead to the deflection of electrons. Following the right-hand rule, the electron beams, irradiated on the left and right domains with opposite in-plane magnetization orientations, are deflected in left and right directions, respectively. When imaging the domains under in-focus conditions, these deflecting electrons are focused in the final image plane so that no magnetic contrast appears (Supplementary Figure 1a). By contrast, when the Lorentz TEM is under the over-focus conditions, the electron deflection induces a low intensity contrast since the electrons are deflected away from the domain wall. This results in the appearance of a dark contrast line in the domain wall region (supplementary Figure 1b). Similarly, a bright contrast line appears in under-focus conditions due to the increased electron density caused by the overlap (Supplementary Figure 1c). In this sense, the inversion of the magnetic contrast in the domain wall is observed between the over- and under-focus images. This is a common feature in the Fresnel model images. It is worth noticing that the out-of-plane magnetic components can not affect the electron propagation so that Lorentz TEM cannot detect the out-of-plane magnetic components. In this study, we changed the objective lens current to control the magnetic fields applied to the specimen along the z-axis.

From the above discussion that the magnetic contrasts in the domains wall depend on the defocused conditions it is possible to reconstruct and map the in-plane magnetic components distribution of the domain walls. For this purpose, a commercial software package QPt is used [4-5], where three Lorentz TEM images at different defocus value (under-, in-, and over-focus) were analyzed by using the transport-of-intensity equation (TIE).

$$\frac{2\pi}{\lambda}\frac{\partial I(x,y)}{\partial z} = -\nabla_{\mathrm{xy}}.\left[I(x,y)\nabla_{\mathrm{xy}}\phi(x,y)\right] \tag{1}$$

where $I(x,y)$ and $\phi(x,y)$ stand for the intensity and phase distributions of propagating wave distribution, respectively. $\lambda$ is the electron wavelength. On the other hand, according to Maxwell-Ampére equations, $\phi(x,y)$ and magnetization $\mathbf{m}$ have a relationship

$$\mathbf{m} \times \mathbf{n} = -\frac{\hbar}{et}\phi(x,y) \quad (2)$$

where $e$, $\hbar$ and $t$ are the electron charge, the reduced Planck constant and the thickness of the sample, respectively. $\mathbf{n}$ is the unit vector parallel to the beam direction. The in-plane magnetic components can be obtained as the phase shift $\phi(x,y)$ is known. The intensity gradient $\partial I/\partial z$ can be approximately expressed as $\Delta I/\Delta z$, considering that defocus step $\Delta z$ is far less than focal length. In this study, a typical process to obtain the magnetic components of skyrmion phase is shown in Supplementary Figure 2.

**Supplementary Note 2. Fabrication and characteristic of FeGe nanostripes.**

We fabricated the FeGe nanostripes by a top-down method from the bulk sample. Supplementary Figure 4 schematically illustrates the whole process, which is described as follows

**Step 1**: Following the standard TEM specimen preparation procedure [7], a homogeneous FeGe thin narrow membrane with the desired thickness is carved on the surface of a FeGe bulk by focused ion beam (FIB) milling. The amorphous surface layer induced by the high energy FIB gallium beam is then reduced to about ~2 nm by polishing the surface with the low energy Ga beam.

**Step 2**: Using the Gas Injection System (GIS), an amorphous PtCx film was deposited on either side of the membrane by means of e-beam evaporation. These PtCx layers are nonmagnetic and only used to reduce the Fresnel fringes at the edges, as discussed later. Hence, they have no effect on the magnetic properties of the FeGe nanostripe.

**Step 3**: Using the FIB, the sandwich structure PtCx/FeGe/PtCx is carved to U-shape to prepare the lift-out.

**Step 4**: Using the Omniprobe 200+Micromanipulator, the membrane with two PtCx coating layers is released from the bulk and then transferred onto a clean silicon substrate.

**Step 5**: Using the standard TEM specimen preparation method again, a PtCx/FeGe/PtCx sandwich structure is fabricated by FIB milling.

**Step 6**: Using the FIB, the desired sample is carved to U-shape to prepare the lift-out.

**Step 7**: Using the Micromanipulator, the PtCx/FeGe/PtCx sandwich structure was attached to a TEM Cu chip for the final Lorentz observations.

By adjusting the parameters in the sample preparation process, a variety of TEM specimens are achieved. Parameters of FeGe nanostripes for Lorentz TEM imaging are shown in Supplementary Table 1

**Supplementary Note 3. Comparison of Fresnel fringes at FeGe/PtCx and FeGe/Vacuum interfaces.**

In transmission electron microscopy, Fresnel contrast occurs if the observed region in which the projected electromagnetic potentials - either mean inner potential or magnetic potential or thickness - changes abruptly and is imaged under out of focus conditions [8]. The Fresnel fringe contrast is often seen at the edge of an object imaged under out-of-focus. For magnetic characterization, the Fresnel imaging technique, as discussed in Supplementary Note 1, enables direct observation of

magnetic structure e.g. domain wall, provided that the thickness variation or projected electrostatic potentials are negligibly small as compared with the magnetic induction contributions [9]. However, at specimen edges, variation in Fresnel fringes due to the abrupt change in thickness overshadows the contrast change due to magnetic potential. This makes the analysis of magnetic information at the edge extremely difficult. Previous Lorentz TEM investigation on FeGe thin plates has illustrated the artificial magnetic contrast due to the Fresnel fringes extends above ~100 nm [5]. Concerning the helical period of FeGe, it is ~ 70 nm. This is sufficient to completely eradicate or severely distort the real domain structure of the edge of interest.

In this study, the use of amorphous PtCx adjacent to the edge of FeGe greatly reduces the effect of Fresnel fringes at the specimen edges due to the much-reduced variation in thickness, as opposed to the case of vacuum-edge. A direct comparison of Fresnel fringes at FeGe/PtCx and FeGe/vacuum interfaces is illustrated in Supplementary Figure 5. For this FeGe nanostripe, the PtCx layers were first coated on two sides of the stripe, and the right side PtCx layer falls off by chance. This thus enables us to directly compare the influence of the Fresnel fringes at the two FeGe/PtCx and FeGe/vacuum interfaces. Supplementary Figures 5a, b, and c show the TEM images of the two interfaces under in-, over- and under-focus conditions, respectively. The yielded magnetic components are shown in Supplementary Figure 5d, where the significantly reduced Fresnel effect at the FeGe/PtCx interface is clearly observed. It is therefore possible to investigate the magnetic helix and skyrmion states in geometrically confined nanostructure by real-space Lorentz microscopy observation.

**Supplementary Note 4. Analysis of the magnetic contrast around the edge.**

Here we present the edge state in a 305 nm nanostripe (see Supplementary Figure 9) an enlarged scale to permit closer inspection of the detailed spin arrangements around the edge. Supplementary Figure 7a shows the spin texture at zero magnetic field, where a periodically modulated half-disk domains, marked by white (clockwise) and red (counter-clockwise) arrows, is clearly observed. This observation is the common feature in the nanostripes only if $\mathbf{Q} \parallel \text{edge}$. It is noteworthy that the FeGe/PtCx interface (Supplementary figure 5) still leads to weak artificial magnetic contrasts though its strength and extended regions are significantly reduced. As a result, the observed magnet components around the nanostripe edge are the superposition of the real and weak artificial ones. To analyze the real magnetic structures, we performed the Lorentz TEM measurement on the nanostripe in the same region at $T$ ~ 300 K. Since the temperature ~ 300 K is far above the Curie temperature $T_c$ ~ 280 K of the FeGe sample [10], the magnetic contrast of the edge comes solely from the edge Fresnel fringes. Supplementary Figure 7b shows the yielded artificial magnetic arrangements around the edge of the nanostripe, where the orientation of the moments is along the right direction (marked by red arrows). This orientation is consistent with the counter-clockwise half-disk domains. This means the real counter-clockwise half domains are strengthened by the artificial magnetic contrast. Meanwhile, the clockwise ones are weakened. A simple assumption is that these half domains are degenerate since no extra effects are able to break the degeneracy at zero magnetic field. This assumption is supported by the model calculations [11]. Accordingly, we proposed the real magnetic structure with

degenerate half domains around the edges (see Supplementary Figure 7c).

When the magnetic field is applied, the degenerate edge state is broken by forming the skyrmions in the interior (Figure 1 in the main text). In this case, a uniform edge state appears. According to the theoretical prediction that a magnetic field will weaken the edge lateral magnetization, but cannot fully polarize the edge spins even at the highest magnetic field due to the boundary conditions [12-14]. To test this prediction, we plotted the lateral magnetization of the uniform edge state as a function of the external magnetic field. By roughly measuring the arrows length around the edge, we clearly observed that the lateral magnetization of the uniform edge state decreases with increasing magnetic field (Supplementary Figure 8e), which is in accordance with the theory. On the other hand, the above-mentioned artificial magnetic contrast contributes to the observed magnetic contrast. For comparison, the artificial magnetization is also plotted by the open star. It shows the real in-plane magnetic components cannot be well distinguished from the artificial magnetic contrast at high magnetic field.

## Supplementary References


[1] Bauer, A., and Pfleiderer, C. Magnetic phase diagram of MnSi inferred from magnetization and ac susceptibility. *Phys. Rev. B* **85**, 214418 (2012)

[2] Meynell, S. A., Surface twist instabilities and skyrmion states in chiral ferromagnets. *Phys. Rev. B* **90**, 014406 (2014)

[3] Beaulieu, D. C., Electron beam chemical vapor deposition of platinum and carbon. The thesis for degree master. (2005)

[4] Yu, X. Z. *et al.* Real-space observation of a two-dimensional skyrmion crystal. *Nature* **465**, 901-904 (2010)

[5] Yu, X. Z. *et al.* Near room-temperature formation of a skyrmion crystal in thin-films of the helimagnet FeGe. *Nature Mater.***10**, 106-109 (2011)

[6] Chapman, J. N., The investigation of magnetic domain structures in thin foils by electron microscopy. *J. Phys. D: Appl. Phys.*, **17,** 623-647 (1984)

[7] Mayer, J., Giannuzzi, L. A., Kamino, T., and Michael, J. TEM sample preparation and FIB-induced damage. *MRS Bulletin*. **32**, 400-407 (2007)

[8] Williams, D. B., and C.B. Carter, C. B., Transmission electron microscopy: A textbook for materials science, Springer, New York, (2009)

[9] Chapman, J. N., and Scheinfein, M. R., Transmission electron microscopies of magnetic microstructures. *J. Magn. Magn. Mater.* **200**, 729-740 (1999)

[10] Wilhelm H., *et al*. Precursor phenomena at the magnetic ordering of the cubic helimagnet FeGe. *Phys. Rev. Lett.***107**, 127203 (2011)

[11] Keesman, R. et al., Néèl skyrmions in confined geometries and at nonzero temperatures. arXiv:1506.00271v1 (2015)

[12] Du, H. F. *et al*. Field-driven evolution of chiral spin textures in a thin helimagnet nanodisk. *Phys. Rev. B* **87**, 014401 (2013)

[13] Leonov, A. O., Rößler, U. K., and Mostovoy, M. Target-skyrmions and skyrmion clusters in nanowires of chiral magnets. *EPJ Web of Conference*s **75**, 05002 (2014)

[14] Rohart, S. and Thiaville, A. Skyrmion confinement in ultrathin film nanostructures in the presence of Dzyaloshinskii-Moriya interaction. *Phys. Rev. B* **88**, 184422 (2013)
